# Activation of Antioxidative Functions by Radon Inhalation Enhances the Mitigation Effects of Pregabalin on Chronic Constriction Injury-Induced Neuropathic Pain in Mice

**DOI:** 10.1155/2016/9853692

**Published:** 2015-12-21

**Authors:** Takahiro Kataoka, Shunsuke Horie, Reo Etani, Norie Kanzaki, Kaori Sasaoka, Yusuke Kobashi, Katsumi Hanamoto, Kiyonori Yamaoka

**Affiliations:** Graduate School of Health Sciences, Okayama University, 5-1 Shikata-cho, 2-chome, Kita-ku, Okayama-shi, Okayama 700-8558, Japan

## Abstract

Radon inhalation brings pain relief for chronic constriction injury- (CCI-) induced neuropathic pain in mice due to the activation of antioxidative functions, which is different from the mechanism of the pregabalin effect. In this study, we assessed whether a combination of radon inhalation and pregabalin administration is more effective against neuropathic pain than radon or pregabalin only. Mice were treated with inhaled radon at a concentration of 1,000 Bq/m^3^ for 24 hours and pregabalin administration after CCI surgery. In mice treated with pregabalin at a dose of 3 mg/kg weight, the 50% paw withdrawal threshold of mice treated with pregabalin or radon and pregabalin was significantly increased, suggesting pain relief. The therapeutic effects of radon inhalation or the combined effects of radon and pregabalin (3 mg/kg weight) were almost equivalent to treatment with pregabalin at a dose of 1.4 mg/kg weight or 4.1 mg/kg weight, respectively. Radon inhalation and the combination of radon and pregabalin increased antioxidant associated substances in the paw. The antioxidant substances increased much more in radon inhalation than in pregabalin administration. These findings suggested that the activation of antioxidative functions by radon inhalation enhances the pain relief of pregabalin and that this combined effect is probably an additive effect.

## 1. Introduction

Radon therapy is performed for mainly pain-related diseases using radon hot springs in Japan [1] and Europe [2] and using mines in Europe [2]. In both cases, patients inhale radon through their nose because radon is a radioactive gas. The conditions of radon therapy in the Misasa Medical Center in Japan and the hospitals in Europe are slightly different [3]. The radon concentration in the treatment room of the Misasa Medical Center is approximately 2,000 Bq/m^3^ [1], while the concentration in Europe is twenty-five times higher [2]. However, the beneficial effects of radon therapy show that there is not much difference between Misasa and Europe in terms of alleviation of pain-related diseases. A report suggested that radon and thermal therapy using hot springs alleviated osteoarthritis [1]. One of the possible reasons for the effects is the prevention of peroxidation reactions and immune depression. Another possible reason is an increase in tissue perfusion because vasoactive-associated substance decreased and pain-associated substance increased by radon therapy. Another report suggested that not only radon and thermal therapy, but also thermal therapy alleviated ankylosing spondylitis [4]. However, radon and thermal therapy continue the alleviation effect longer than thermal therapy. Radon therapy is also effective against rheumatic diseases [5]. In addition, radon therapy reduced the dosage of medicine [6]. These findings indicated that radon therapy has a positive effect on pain-related diseases such as osteoarthritis, ankylosing spondylitis, and rheumatic diseases. Although it is likely that radon therapy has a positive effect against pain-related diseases, it is insufficient to understand the mechanisms of radon therapy. To investigate the mechanisms, we previously reported that radon inhalation has preventive and curative effects against chronic constriction injury (CCI) in mice due to the reduction of reactive oxygen species (ROS) by the activation of antioxidative functions following radon inhalation [7].

Pregabalin is a medicine that relieves pain. Toth reviewed the clinical implications for the management of neuropathic pain [8]. Clinical studies suggested that administration of pregabalin is effective against diabetic peripheral neuropathy. But the doses of up to 150 mg/day are inefficacious [9]. Several adverse central nervous system effects, such as dizziness and somnolence, and adverse systemic effects, such as peripheral edema, were observed [8]. The incidence of these adverse effects increases with larger pregabalin doses [8, 10].

It is well known that radon inhalation increases lung cancer risk [11]. Therefore, a combination of pregabalin and radon treatment has beneficial effects to reduce the adverse effects of pregabalin and the lung cancer risk caused by radon inhalation. The purpose of this study was to compare the mitigating effects on CCI of radon inhalation and pregabalin administration and to examine the combination effects of radon and pregabalin on CCI-induced neuropathic pain. We assayed the behavioural response to evaluate the pain and the following biochemical parameters to assess the effects of radon and pregabalin treatment: SOD activity, catalase activity, total glutathione content (t-GSH), and lipid peroxide level in paw.

## 2. Materials and Methods

### 2.1. Animals

Male ICR mice (age, 8 weeks; body weight, approximately 33–40 g) were obtained from Charles River (Yokohama, Japan). Ethical approval for all protocols and experiments was obtained from the Animal Experimentation Committee of Okayama University. The mice were housed under a 12:12 h artificial light cycle (8:00 a.m. to 8:00 p.m.) at a temperature of 22 ± 2°C.

### 2.2. Behavioral Testing: von Frey Test

The behavioral response of mice to mechanical stimuli was assessed using the von Frey test [12]. von Frey tests were conducted once a day before (2 or 3 days) and after (2 or 3 days) CCI surgery and at 30, 60, 90, and 120 min after pregabalin administration, 24-hour radon inhalation, or a combination of radon inhalation and pregabalin administration. In the group treated with radon and pregabalin, mice were administered pregabalin immediately after radon inhalation. Mice were individually placed in plastic cages with a wire-mesh floor (1 mm diameter wire placed 5 mm apart). The paw was touched with 1 of a series of 9 von Frey hairs (0.04, 0.07, 0.16, 0.4, 0.6, 1, 1.4, 2, and 4 g) (North Coast Medical Inc., CA, USA). A positive response was noted when the paw was sharply withdrawn. The 50% withdrawal threshold was determined using the up-down method [12]. Briefly, the behavioral test was initiated with the 0.6 g von Frey hair, representing the middle of the series. A stronger stimulus was chosen if the paw showed a negative response. A weaker stimulus was chosen if the paw showed a withdrawal response. The critical 6 data points were noted after the response threshold was first crossed.

50% g threshold = 10^*X*_*f*_+*kδ*^, where *X*_*f*_ is log units value of the final von Frey hair used; *k* is tabular value for the pattern of positive or negative responses [13]; and *δ* is mean difference (in log units) between stimuli.

### 2.3. CCI Surgery

Mice received a unilateral CCI after a pretest for mechanical sensitivity using a von Frey test. Briefly, the right sciatic nerve was exposed at the midthigh level and was then constricted loosely with three ligations using 4-0 chromic gut, each spaced about 1 mm apart under sodium pentobarbital anesthesia (50 mg/kg, i.p.). The same surgical operation was performed, without CCI, on a group of animals that served as sham-operated controls.

### 2.4. Radon Inhalation

**Figure 1:**
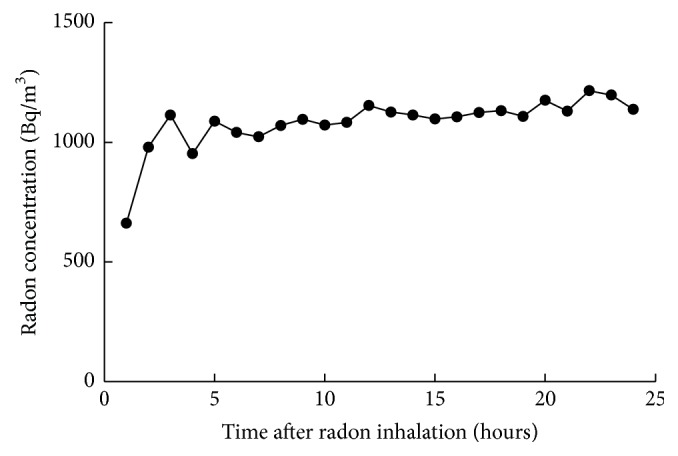
Changes in radon concentration in the mouse cage.

To generate conditions for inhalation of specified radon concentrations, our radon exposure original system was used as described in a previous report [14]. The radon concentration in the mouse cage was measured using a radon monitor (CMR-510, femto-TECH Inc., Ohio, USA). The mean concentration of radon was approximately 1,000 Bq/m^3^ (Figure 1). The mice inhaled radon at a concentration of 1,000 Bq/m^3^ for 24 hours after CCI while having free access to food and water during radon inhalation.

### 2.5. Pregabalin Treatment

Pregabalin (1, 3, or 10 mg/kg body weight; Sigma-Aldrich Japan Co. LLC., Tokyo, Japan) was injected into the peritoneum of the mice after the CCI operation. To examine the combination effects of radon and pregabalin, mice were administered pregabalin at a dose of 3 mg/kg of body weight immediately after radon inhalation.

### 2.6. Biochemical Assays

Paws were homogenized on ice in 10 mM phosphate buffer (PBS; pH 7.4). The homogenates were used for the assays of SOD and catalase.

SOD activity was assayed by the nitroblue tetrazolium (NBT) reduction method using the Wako-SOD test (Wako Pure Chemical Industry, Co., Ltd., Osaka, Japan) [15]. Briefly, the homogenates were centrifuged at 12,000 ×g for 45 min at 4°C and the supernatants were used to assay SOD activity. SOD activity in the paw was measured by the extent of inhibition of the reduction in NBT measured at 560 nm using a spectrophotometer. One unit of enzyme activity was defined as 50% inhibition of NBT reduction.

Catalase activity was measured as the hydrogen peroxide (H_2_O_2_) reduction rate at 37°C and was assayed at 240 nm using a spectrophotometer [16]. The assay mixture consisted of 50 *μ*L of 1 M Tris-HCl buffer containing 5 mM ethylenediaminetetraacetic acid (pH 7.4), 900 *μ*L of 10 mM H_2_O_2_, 30 *μ*L deionized water, and 20 *μ*L paw supernatant. Activity was calculated using a molar extinction coefficient of 7.1 × 10^−3 ^M^−1 ^cm^−1^. The changes of absorbance were observed for a minute.

T-GSH content was measured using the Bioxytech GSH-420 assay kit (OXIS Health Products, Inc., Portland, OR, USA). Briefly, tissue samples from the paw were homogenized in 10 mM PBS (pH 7.4) and then mixed with ice-cold 7.5% trichloroacetic acid solution. The homogenates were centrifuged at 3,000 ×g for 10 min. Assays were performed on tissue supernatants. This assay is based on the formation of a chromophoric thione, the absorbance of which can be measured at 420 nm and is directly proportional to the t-GSH concentration.

Lipid peroxide levels were assayed using the Bioxytech LPO-586 assay kit (OXIS Health Products, Inc.). Briefly, the paw samples were homogenized in 10 mM phosphate buffer (PBS; pH 7.4) on ice. Prior to homogenization, 10 *μ*L of 0.5 M butylated hydroxytoluene in acetonitrile was added per 1 mL of the buffer-tissue mixture. After homogenization, the homogenate was centrifuged at 15,000 ×g, for 10 min at 4°C, and the supernatant was used for the assay. The lipid peroxide level assay is based on the reaction of a chromogenic reagent, N-methyl-2-phenylindole, with malondialdehyde and 4-hydroxyalkenals at 45°C. The optical density of the colored products was read at 586 nm in a spectrophotometer.

The protein content in each sample was measured by the Bradford method, using the Protein Quantification Kit-Rapid (Dojindo Molecular Technologies, Inc., Kumamoto, Japan) [17].

### 2.7. Statistical Analyses

The data are presented as the mean ± standard error of the mean (SEM). Each experimental group consisted of samples from 5-6 animals. Statistically significant differences were determined using an unpaired *t*-test for comparisons between two groups and Tukey's tests for multiple comparisons where appropriate. *P* values were considered significant at *P* < 0.05.

## 3. Results

### 3.1. Effect of Sham-Operation, Radon Inhalation, and Pregabalin Administration on 50% Paw Withdrawal Threshold following CCI Surgery

**Figure 2:**
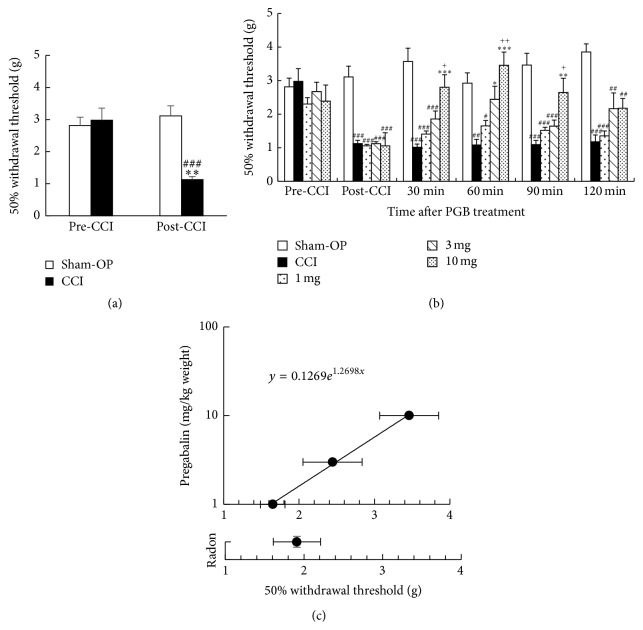
Changes in 50% paw withdrawal threshold following pregabalin (PGB) administration. (a) Changes in sham-operation (Sham-OP) or CCI surgery on 50% paw withdrawal threshold. ^*∗∗*^ *P* < 0.01 versus pre-CCI (CCI), ^###^ *P* < 0.001 versus post-CCI (Sham-OP). (b) Time- and dose-dependent changes of 50% paw withdrawal threshold. ^*∗*^ *P* < 0.05, ^*∗∗*^ *P* < 0.01, and ^*∗∗∗*^ *P* < 0.001 versus each CCI, ^#^ *P* < 0.01, ^##^ *P* < 0.01, and ^###^ *P* < 0.01 versus each sham-OP, and ^+^ *P* < 0.05, ^++^ *P* < 0.01 versus each 1 mg. (c) Effectiveness comparison between radon and pregabalin. Values are presented as the mean ± SEM of data from 5-6 animals.

We first confirmed whether the sham-operation decreased the 50% paw withdrawal threshold or not. Results showed that the sham-operation did not decrease the 50% paw withdrawal threshold whereas CCI surgery significantly decreased it (Figure 2(a)).

Next, we examined dose- and time-dependent changes in the 50% paw withdrawal threshold following pregabalin administration. Results showed that the peak of the mitigative effects was at around one hour after pregabalin administration. In addition, mechanical allodynia was mitigated in a dose-dependent manner at 60 minutes after pregabalin administration (Figure 2(b)).

Then, we compared the mitigative effects of radon inhalation and pregabalin administration at one hour after treatment of radon or pregabalin because the peak of mitigative effects is at around one hour after pregabalin administration. As a result, radon inhalation has a mitigating effect against mechanical allodynia similar to the effects of approximately 1.4 mg/kg of body weight of pregabalin (Figure 2(c)).

### 3.2. Effect of Pregabalin Administration on Antioxidative Functions in Paw

To clarify the changes in the antioxidant associated substances after pregabalin administration, SOD, catalase, t-GSH, and lipid peroxide level were assayed.

**Figure 3:**
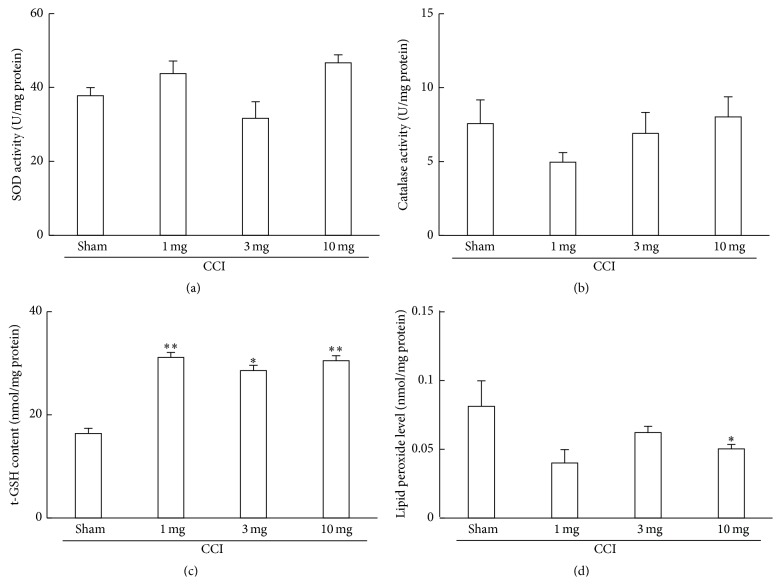
Changes in antioxidant-associated parameters in the paw following CCI surgery and pregabalin administration. Sham; no pregabalin administration. Values are presented as the mean ± SEM of data from 5-6 animals. ^*∗*^ *P* < 0.05, ^*∗∗*^ *P* < 0.01 versus sham (CCI only; no administration of pregabalin).

Although no significant changes were observed in the activities of SOD and catalase, pregabalin administration increased t-GSH content in paw. The lipid peroxide level in the paw of mice, which were administrated pregabalin at a dose of 10 mg/kg of body weight, significantly decreased (Figure 3).

### 3.3. Combined Effects of Radon and Pregabalin on CCI-Induced Neuropathic Pain

To clarify the combined effects of radon and pregabalin on neuropathic pain, von Frey tests were conducted.

**Figure 4:**
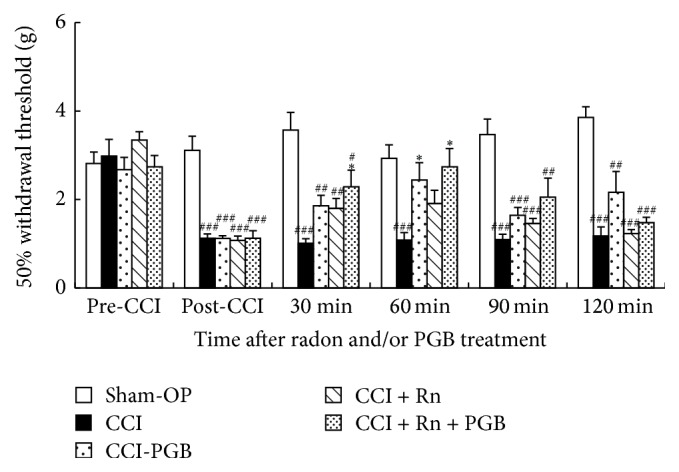
Effectiveness comparison among radon (Rn), pregabalin, and combination of radon and pregabalin. Sham-OP: sham-operation, PGB—3 mg/kg weight, Rn—1,000 Bq/m^3^, and Rn + PGB—1,000 Bq/m^3^ + 3 mg/kg weight. Values are presented as the mean ± SEM of data from 6 animals. ^*∗*^ *P* < 0.05 versus each CCI, ^##^ *P* < 0.01, and ^###^ *P* < 0.01 versus each sham-OP.

The fifty percent paw withdrawal threshold was significantly decreased by CCI surgery. The 50% paw withdrawal threshold of mice treated with pregabalin (60 min) or radon and pregabalin (30 min, 60 min) was significantly increased. Radon inhalation also increased the 50% paw withdrawal threshold, but this difference was not significant (Figure 4).

From the formula in Figure 2(c), we estimated the combined effects of radon and pregabalin (3 mg/kg of body weight) on CCI-induced neuropathic pain. As a result, the combination of radon and pregabalin has a mitigative effect against mechanical allodynia similar to the effects of approximately 4.1 mg/kg of body weight of pregabalin. Therefore, this combined effect is probably an additive effect because of the mitigating effect of radon similar to the effects of approximately 1.4 mg/kg of body weight of pregabalin.

### 3.4. Effect of Sham-Operation, Radon Inhalation, and Pregabalin Treatment on Antioxidative Functions in Paw

To clarify the involvement of the antioxidant effects, antioxidant associated substances, such as SOD, catalase, t-GSH, and lipid peroxide level were assayed.

**Figure 5:**
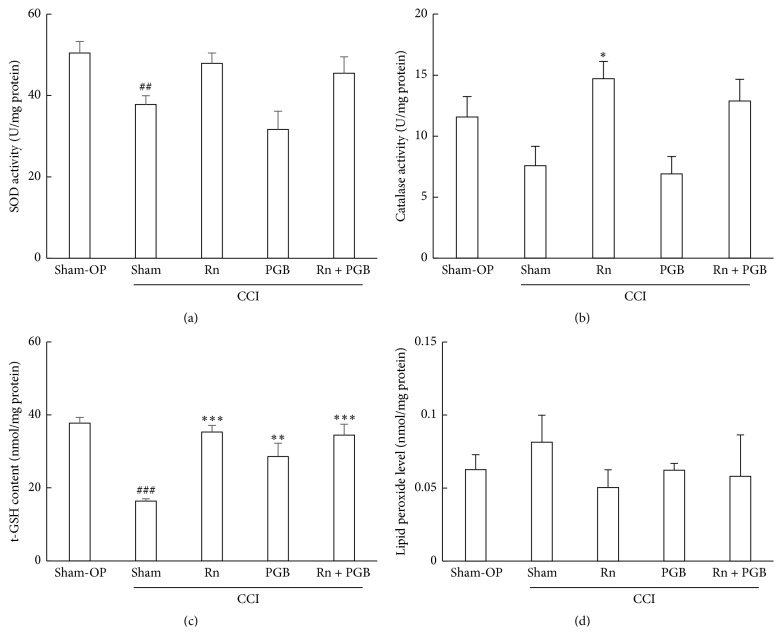
Changes in antioxidant-associated parameters in the paw. Values are presented as the mean ± SEM of data from 6 animals. ^*∗*^ *P* < 0.05, ^*∗∗*^ *P* < 0.01, and ^*∗∗∗*^ *P* < 0.001 versus sham (CCI only; no treatment with either radon or pregabalin, pregabalin; 3 mg/kg weight); ^##^ *P* < 0.01, ^###^ *P* < 0.001 versus sham-OP.

SOD activity in paw of mice that received CCI surgery significantly decreased. The SOD activity of mice administered pregabalin following CCI surgery was at the same level as that of mice that had received CCI surgery. However, the SOD activities of radon or radon and pregabalin treated mice were at the same level as that of sham-operated mice (Figure 5).

Catalase activity in the paw of mice that received CCI surgery decreased, but this difference was not significant. The catalase activity of mice administered pregabalin following CCI surgery was at the same level as that of mice that had received CCI surgery. However, the catalase activities of radon inhaled mice were significantly increased compared with CCI surgery received mice. In addition, the catalase activity of radon and pregabalin treated mice was at the same level as that of radon treated mice (Figure 5).

The t-GSH content in the paw of mice that had received CCI surgery significantly decreased. The t-GSH contents of mice treated with pregabalin, radon, or radon and pregabalin following CCI surgery were significantly increased compared with that of CCI surgery received mice (Figure 5).

The lipid peroxide level in the paw of mice that had received CCI surgery increased, but this difference was not significant. The lipid peroxide levels of mice treated with pregabalin, radon, or radon and pregabalin following CCI were lower than that of CCI surgery received mice (Figure 5).

## 4. Discussion

The possible mechanisms of action for pregabalin are not completely understood [8]. It has been reported that pregabalin binds with high affinity to the calcium channel alpha2-delta (Ca_V_ *α*_2_-*δ*) site [8]. Reducing the stimulated synaptic influx of calcium reduces the stimulated release of transmitters such as glutamate, noradrenaline, GABA, and acetylcholine at the neuromuscular junction and spinal inhibitory glycine [18]. To our knowledge, there are no reports that low-dose irradiation including radon inhalation has effects similar to pregabalin as described above or that pregabalin has antioxidative effects. Therefore, we assumed that the combination of radon inhalation and pregabalin administration has additive or synergetic effects on CCI-induced neuropathic pain. In this study, we focused on the antioxidative functions.

Low-dose X- or *γ*-irradiation has a stimulatory effect on animals, and the stimulation induced the activation of antioxidative functions [19, 20] and immune functions [21, 22]. These activations contribute to the inhibition of oxidative stress induced damages. For example, continuous low-dose-rate *γ*-irradiation ameliorates diabetic nephropathy in mice through the activation of antioxidative functions in the kidney [23]. Another report suggested that low-dose *γ*-ray irradiation attenuates collagen-induced arthritis by suppressing proinflammatory cytokines and autoantibody production and by inducing regulatory T cells [24]. Although radon is a gas and emits *α*-ray, similar effects were observed. Radon inhalation increases SOD activity in many organs of mice [25] and inhibits some kinds of oxidative damage [3]. However, the estimated absorbed doses of these organs by radon inhalation are much smaller than those by X- or *γ*-irradiation [26]. To clarify the radon effects, future research is required because there are no data to explain why radon inhalation increases antioxidative functions.

Oxidative stress is involved in the neuropathic pain conditions. For example, N-acetyl-L-cysteine (NAC), which acts as a cysteine donor, resulted in significant reduction of hyperalgesia in CCI-induced neuropathic pain in rats. This report also suggested that glutathione plays an important role in the inhibition of neuropathic pain because of its capacity to donate cysteine amino acid, a component of glutathione [27]. We previously reported that radon inhalation brings pain relief for CCI-induced neuropathic pain in mice due to the activation of antioxidative functions [7]. This activation is involved in the decrease in the inflammatory leukocytes migration in the paw, indicating the anti-inflammatory effects of radon inhalation. These findings suggest that inhibition of overproduction of ROS plays an important role in the mitigation of CCI-induced neuropathic pain. In this study, antioxidant associated substances were increased by radon inhalation and 50% paw withdrawal thresholds were increased. Interestingly, antioxidant associated substances of mice treated with a combination of radon and pregabalin were increased. Although the t-GSH of mice treated with pregabalin increased, the activities of SOD and catalase did not increase. These findings suggested that antioxidative functions activate much more in radon inhalation than pregabalin administration and that the mitigation mechanisms of radon and pregabalin are different.

Our previous report suggested that radon inhalation at a concentration of 2,000 Bq/m^3^ is more effective in CCI-induced neuropathic pain in mice than that of 1,000 Bq/m^3^ [7]. In this study, we made a choice of a lower radon concentration because lower radon concentration can reduce the absorbed dose. The absorbed dose from radon inhalation should be reduced to reduce lung cancer risk. In this study, the combination of radon and pregabalin enhanced the mitigative effect against mechanical allodynia. These findings indicate the usefulness of the combination of radon and pregabalin.

In conclusion, radon inhalation at a concentration of 1,000 Bq/m^3^ for 24 hours has a mitigative effect against mechanical allodynia similar to the effects of approximately 1.4 mg/kg weight of pregabalin. The combined effect of radon and pregabalin is an additive effect because the combination of radon and pregabalin has a mitigative effect against mechanical allodynia similar to the effects of approximately 4.1 mg/kg weight of pregabalin. The possible mechanism of the additive effect is the activation of antioxidative functions induced by radon inhalation. However, the effects of radon inhalation on the nerve system have yet to be confirmed. They provide a substantial basis for future studies aimed at assessing the detailed mechanisms of the additive effects of neuropathic pain.
